# Enhanced oral bioavailability of oligomeric proanthocyanidins by a self‐double‐emulsifying drug delivery system

**DOI:** 10.1002/fsn3.1673

**Published:** 2020-06-01

**Authors:** Yuan Tian, Xinyu Mao, Rui Sun, Ming Zhang, Qiang Xia

**Affiliations:** ^1^ School of Biological Sciences and Medical Engineering Southeast University Nanjing China; ^2^ State Key Laboratory of Bioelectronics School of Biological Sciences and Medical Engineering Southeast University Nanjing China

**Keywords:** bioavailability, oligomeric proanthocyanidin, self‐double‐emulsifying drug delivery system, solid form, stability

## Abstract

The present study aims at the formulation and evaluation of solid self‐double‐emulsifying drug delivery system (SDEDDS) to increase the bioavailability of oligomeric proanthocyanidin (OPC). The formulation is prepared through two‐step method and is able to form water‐in‐oil‐in‐water (W/O/W) double emulsions after diluted with aqueous medium while keeping the drug in inner water phase. Solid‐state characterization is performed by DSC and X‐ray powder diffraction. Furthermore, antioxidant capacity shows that OPC is preserved by the solid SDEDDS. OPC‐SDEDDS exhibit sustained release of OPC under the conditions mimicking gastrointestinal tract. The result shows that bioaccessibility of OPC is improved after incorporating into SDEDDS formulation compared to pure drug. The proposed SDEDDS is a promising carrier strategy for delivering the hydrophilic compounds with low‐oral bioavailability.

## Graphical abstract

This study aimed at the formulation and evaluation of self‐double‐emulsifying drug delivery system (SDEDDS) for the application in dietary supplement of oligomeric proanthocyanidin (OPC). It also demonstrates that the novel SDEDDS in solid form could be a promising formulation strategy for the drug delivery of hydrophilic compounds with low‐oral bioavailability.

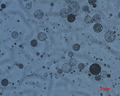

## 1 INTRODUCTION

The natural bioactive proanthocyanidin (PC), which is widely found in plants, could be derived from polymerization of flavanol monomers. Its monomer includes catechin, epicatechin, epigallocatechin, and epicatechin gallate which forms a dimer to multimer by polymerization (Lelone & Tachibaba, 2013). When the degree of monomer polymerization is between 2 and 4, it would aggregate into oligomeric proanthocyanidin (OPC). OPC has a wide range of pharmacological properties, which is well‐known as one of the most effective free radical scavengers and lipid peroxidation inhibitors that has been discovered. In addition, the OPC gains anticancer activity (Huynh & Teel, 2000), anticardiovascular activity (Wegrowski, Robert, & Moczar, 1984), anti‐allergic activity (Sen & Bagchi, 2001), enzyme inhibitory activity (An & Choi, 1994), and anti‐ulcer activity (Saito, Hosoyama, Ariga, Kataoka, & Yamaji, 1998).

Notwithstanding, OPC has been found with multiple beneficial effects. Its in vivo efficacy is controversial due to the low bioavailability. Since OPC is easily oxidized when exposed to air and converts to anthocyanins when heated in acidic solution (Deprez, Mila, Huneau, Tom, & Scallbert, 2001), the instability dominates the low bioavailability of OPC. Thus, OPC would be classified as biopharmaceutics classification system (BCS) class III: high solubility and low permeability (Vertzoni, Pastelli, Psachoulias, Kalantzi, & Reppas, 2007).

The double emulsion (Matsumoto, Kohda, & Murata, 1977), which has been known from late 1960s, is a complicated and heterogeneous system combining two types of emulsion at the same time. The double emulsion can protect hydrophilic bioactive ingredients from degradation and penetrate the small intestinal epithelium. This technique has been broadly used to increase the absorption of bioactive compounds in oral administration (Koga, Takarada, & Takada, 2010; Shima et al., 2006). Nevertheless, double emulsion has thermodynamically instability with a strong tendency toward coalescence, flocculation, and creaming. These adverse conditions limit the industrial application on it (Lutz, Aserin, Wicker, & Garti, 2009). Despite many efforts to stabilize of double emulsion, few have overcome the research state and been marketing successfully (Matos, Gutierrez, Coca, & Pazos, 2014; Schuch, Deiters, Henne, Kohler, & Schuchmann, 2013; Tang & Sivakumar, 2012). Thereby the self‐double‐emulsifying drug delivery system (SDEDDS) was developed to improve stability and enhance the oral bioavailability (Lv et al., 2012).

The water‐in‐oil (w/o) emulsion and the hydrophilic surfactant constitute the SDEDDS and can be spontaneously emulsified in the aqueous media to form w/o/w double emulsions (Qi, Wang, Zhu, Hu, & Zhang, 2011). However, most of traditional SDEDDS are in liquid form and could lead to some flaws such as instability, low convenience, high production costs, and rare choices of dosage forms (Lv et al., 2012). Thus, solidifying SDEDDS become popular. However, the research of OPC‐loaded SDEDDS for oral delivery remains ambiguous. It is essential to develop a solid OPC‐SDEDDS to improve the stability and oral bioavailability of OPC.

Regarding these therapeutic advantages and the physicochemical characteristics of bioactive substance, which is suitable for oral administration, the solid OPC‐SDEDDS is confirmed its optimal formula by utilizing a revised two‐step emulsification way. Physicochemical characterization, in vitro and vivo absorption experiments, and stability evaluation are used for developing OPC‐SDEDDS.

## 2 MATERIALS AND METHODS

### 2.1 Materials

Oligomeric proanthocyanidin (OPC, 98% purity) was obtained from Senfu Biochemical Co., Ltd.; polyglycerol polyricinoleate (PGPR) was purchased from Shanghai Youchuang industrial Co., Ltd.; corn oil, soy oil, sunflower oil, and rapeseed oil were purchased from Qinhuangdao Goldensea Foodstuff Industries Co., Ltd.; Span 60, Span 80, Tween 20, Tween 60, Tween 80, and medium‐chain triglyceride (MCT) were from Sinopharm Chemical Reagent; polyglyceryl‐2distearate (B‐18b) was obtained from Shanghai Intron Food Co., Ltd.; glycerin monostearate (GMS) was supplied by Lonza Group; 1,1‐diphenyl‐2‐picrylhydrazyl (DPPH) free radical is supplied by Tokyo Chemical Industry; and simulated gastric fluid (SGF, pH adjusted to 2.0), simulated intestinal fluid (SIF, pH adjusted to 7.5) with pepsin, bile extract, and pancreatin were purchased from Sigma‐Aldrich (porcine). All other chemical solvents were of analytical grade from Sinopharm Chemical Reagent.

### 2.2 Solubility studies

The solubility of OPC was determined in various excipients, including water (pH 5.0), surfactants (PGPR, Tween 20, Tween 60, and Tween 80), solid lipids, and oils (GMS, MCT, corn oil, soy oil, sunflower oil, rapeseed oil, and olive oil) by shaking flask method (Shete & Patravale, 2013). An excess amount of OPC was added to cap vials that contain various excipients selected, respectively. Then, shaking the vials under 5°C above the melting point in a water bath shaker to make it balanced. The admixtures were spun for 10 min at  9,184 *g* (Beckman Coulter, Allegra X‐22R), and supernatant solution was extracted for analysis. The supernatant was diluted to a suitable concentration with ethanol, and the absorbance was measured UV‐visible spectrophotometer at 280 nm. All tests and analyses were duplicated three times.

### 2.3 Preparation of OPC‐SDEDDS

The OPC‐SDEDDS was formulated via a two‐step method. First, prepared preliminary W/O emulsions as bellowed: PGPR and GMS were dissolved in outer oil phase. At the same time, dissolved OPC into inter water phase and well‐mixed thoroughly and then slowly put them into outer oil phase. This primary W/O blends were heated to 65 ± 2°C via water bath and mixed using IKA RW 20 overhead stirrers under 92 *g* for 15 min. Later, the hydrophilic surfactant was added into primary emulsions and heated up to 50°C at 250 rpm for 15 min. After dispersed in deionized water, the resulting admixture could form W/O/W multiple emulsions by self‐emulsifying.

### 2.4 Emulsifying experiments

Preliminary W/O emulsions were formed by selected oils, hydrophobic surfactants within separate concentrations by procedure mentioned above. Based on emulsion stability index (ESI) (Aditya et al., 2015), the excipient was picked attentively. The mixtures were foster 2 hr under 65°C and document the volume of separated layer from emulsions. To obtain more precise data, all experiments were practiced three times. The ESI equation was calculated by following:

(1) $$\text{ESI}=\frac{V\left(\text{total}\right)-V\left(\text{iw}\right)}{V\left(\text{total}\right)}\times 100\%$$

where *V* (total) is the sum volume of sample, and *V* (iw) is the volume of inner water phase. Subsequently, OPC‐SDEDDS was made up with selective hydrophilic surfactants: Tween 20, Tween 60, and Tween 80 in disparate concentrations by previous procedure. The key factor to form successful hydrophilic surfactant was based on optimal self‐emulsification ability (SA) and encapsulation efficiency (EE).

### 2.5 Self‐emulsification ability (SA) of SDEDDS

The gravimetric method was introduced here to help investigate the self‐emulsification ability of OPC‐SDEDDS (Comunian et al., 2013). OPC‐SDEDDS (0.5 g) was added into distilling flask with 10 ml distilled water, and the mixture was homogenized using a horizontal rotator (HY‐4) at 100 rpm for 15 min under room temperature. The final step should centrifuge the solution at 1,125 *g* for 5 min, transfer 5 ml aliquot of supernatant liquor to a preweighed ceramic dish, and keep it in oven for 60°C till fully evaporation of water. This step was also practiced in triplicate. The self‐emulsification ability was calculated based on:

(2) $$\text{SA}=\frac{\text{final}\;\text{weight}\times 2}{\text{initial}\;\text{weight}}\times 100\%$$

### 2.6 Encapsulation efficiency (EE) and drug loading content

In order to measure encapsulation efficiency (EE), centrifugal ultrafiltration was used to separate the free OPC in the SDEDDS (Fan, Liu, Huang, Li, & Xia, 2014). At first, 0.5 g of solid OPC‐SDEDDS was added dispersedly into 9.0 g of deionized water, and note that, dispersion was placed in the upper chamber of centrifuged tube with an ultrafilter (30 kDa, Millipore) and spun at 13,225 *g* for 30 min at 4°C. The OPC in SDEDDS (total drug) and ultrafiltrate (free drug) was determined by UV‐visible spectrophotometer. EE was calculated from the Equation 3, and drug loading content was calculated from the Equation 4. All experiments were verified in triplicate.

(3) $$\text{EE}=\frac{W\left(\text{total}\right)-W\left(\text{free}\right)}{W\left(\text{total}\right)}\times 100\%$$

(4) $$\text{Drug}\;\text{loading}\;\text{content}=\frac{W\left(\text{total}\right)-W\left(\text{free}\right)}{W\left(\text{total}\right)+W\left(\text{excipients}\right)}\times 100\%$$

where *W* (total) is the total amount drug in OPC‐SDEDDS, *W* (free) is the amount of unentrapped drug, and *W* (excipients) represents the weight of added excipients in the preparation stage.

### 2.7 Droplet microscopic observation

The optical microscope (Nikon Microscope Eclipse 50i 55i, Morrell Instrument Co., Inc.) was used to observe samples. A 0.5 g OPC‐SDEDDS was spread in 9.0 g deionized water beforehand. Afterward, the particles were examined on slides under 100× magnifications with microscope.

### 2.8 Viscosity determination

To measure the viscosity of current SDEDDS formulations, a cone type viscometer (HBDV‐II + P) was used without dilution. The shear rate was ranged from 0.1 to 300 s^−1^ and performed the measurement under room temperature (25 ± 2°C). The statistical mean value of constant shear viscosity was obtained from the data at 300 s^−1^ and expressed as mean ± *SD* (*n* = 3).

### 2.9 X‐ray diffractometry (XRD)

A powder diffractometer (D8 Discover, BRUKER) was utilized for XRD scattering measurements. GMS crystals, OPC, and OPC‐SDEDDS were taken to perform powder XRD analysis. The experiment condition: Copper target Kα ray source had 40 mA current, 40 kV supply voltage, scan angle (2*θ*) between 4° and 60°, step size of 0.02°, and scan rate of 5°/min.

### 2.10 Differential scanning calorimetry (DSC)

Crystallinity curve was tested using differential scanning calorimetry (Mettler Toledo Instruments) for solid OPC‐SDEDDS, OPC, and the physical mixture that made up the excipients in OPC‐SDEDDS, respectively. The analysis condition: An empty aluminum pan was taken as a control. The heating rate was 5°C/min, the scanning temperature range was 5–80°C, and the dry nitrogen flow rate was 40 ml/min.

### 2.11 DPPH radical scavenging activity

The antioxidant activity of solid OPC‐SDEDDS formulations was evaluated by the method proposed by Okonogi and Riangjanapatee with some revisions (Okonogi & Riangjanapatee, 2015). Simply to say, 0.2 mM DPPH ethanol solution, aqueous suspensions of OPC‐SDEDDS with OPC concentrations of 10–100 µg/ml, and OPC ethanol solutions of 10–100 µg/ml were prepared and stored, respectively, in separate brown flask. After blending the samples in each brown flask and incubating in dark for 30 min under room temperature, the absorbency of different samples was measured via UV spectrophotometer at 517 nm. Replace OPC‐SDEDDS aqueous suspension and OPC ethanol solution with distilled water and ethanol, respectively, and later used as a control reference. The equation of radical scavenging activity was evaluated as bellowed:

(5) $$\text{DPPH}\;\text{radical}\;\text{scavenging}\;\text{activity}=\frac{A\left(c\right)-A\left(s\right)}{A\left(c\right)}\times 100\%$$

where *A* (s) was the absorbance of samples, and *A* (c) was the absorbance of controls.

### 2.12 Illumination stability of OPC‐SDEDDS

Illumination stability of OPC‐SDEDDS was measured by following methods: Three samples with the same contents of OPC were prepared—two of them were solid OPC‐SDEDDS, while the other was an aqueous solution of OPC. One of the OPC‐SDEDDS was placed under natural light conditions, in order to took comparison with it, another OPC‐SDEDDS was placed in the dark, and OPC aqueous solution was exposed to the light as well. Store at room temperature (25 ± 2°C) for a period of time, the changes in the appearance of samples were observed on the first, 5th, 10th, 20th, and 30th day, and the remaining content of OPC was determined by UV spectrophotometer, respectively. Each value was expressed as mean ± *SD* (*n* = 3).

### 2.13 Storage stability of OPC‐SDEDDS

Self‐double‐emulsifying drug delivery system stability was performed under ICH Q1A (R2) guidelines (2003). Different OPC‐SDEDDS observation groups were stored in 4°C ± 1/65% RH, 25°C ± 2/65% RH, and 40°C ± 1/65% RH for 1 month. The physical and chemical stabilities of OPC‐SDEDDS were varied time‐dependently (0, 7, 14, and 21 days of storage). The appearance of OPC‐SDEDDS, OPC retention ratio of OPC‐SDEDDS, and encapsulation efficiency of OPC‐SDEDDS were recorded sequentially. The value was expressed in mean ± *SD* (*n* = 3).

### 2.14 In vitro release of OPC from OPC‐SDEDDS

The dialysis bag method was introduced here to measure the drug release from OPC‐SDEDDS (Hu, Zhao, Xia, & Sun, 2015; Li et al., 2009). Freshly prepared phosphate buffers of pH 1.2 and pH 6.8 were prepared as release media, and 2 ml of OPC‐SDEDDS suspension or 2 ml of OPC aqueous solution (contenting same amount of OPC) was put separately in dialysis bags (MWCS 12,000 Da, Biosharp) to investigate their release behavior under different media conditions. The dialysis bags were immersed in a 200 ml release medium and were carried out in water bath at 37°C and 100 rpm. These groups were tested, respectively, with the release medium (pH 1.2 and pH 6.8). The release media (3 ml) was extracted periodically at set intervals and added with an equal amount of the same temperature release medium in the same time, for 6 hr. The release medium taken at different times was centrifuged at 9,184 *g* for 10 min, and the content of OPC in the supernatant was measured and calculated by UV spectrophotometer method. Each experiment was done at least three times.

### 2.15 Evaluation of in vitro digestibility of OPC‐SDEDDS

According to the research in vitro digestion simulation process by Huang, Wang, Li, Xia, and Xia (2018), a little adjustment was implemented based on the specific situation to perform the experiment. The experiment was carried out in water bath at 37°C and 100 rpm. The OPC‐SDEDDS (0.5 g) with different ratios of fat crystals was separately taken and mixed with 10 ml SGF (3.2 mg/ml pepsin dissolved in phosphate solution of pH 1.2). After an hour incubation, the pH of digestive solution was adjusted to 7.0 with 0.5 mol/L NaOH. Afterward, 10 ml SIF (4.76 mg/ml pancreatic lipase and 5.16 mg/ml pig bile extract dissolved in pH 6.8) was poured into the mixture. During the 2 hr of the simulated intestinal digestion course, a PH‐stat titration method was developed to evaluate the amount of fatty acids released from lipids. The PH is kept at 7.0 by adding 0.1 mol/L NaOH mildly. The volume added for NaOH was recorded over time. Another experiment was taken in the same time to measure the solubilization amount of OPC when digested, where the NaOH titration was unneeded. After 10 ml SIF was added, aliquots (0.3 ml) were extracted at certain time intervals and mixed with 40 μl of 4‐BPB methanol solution (0.5 M)—this could prevent further lipolysis. Afterward, the samples were spun around with centrifuge to form two divided layers. The upper layer in centrifuge tube was a transparent micelle phase which was diluted and assayed for OPC by UV spectrophotometer. In addition, the bioaccessibility of OPC was calculated according to the following equation (control group was pure OPC aqueous):

(6) $$\text{Bioaccessibility}=\frac{\text{the concentration of OPC in micelle}}{\text{the concentration of OPC before digestion}}\times 100\%$$

### 2.16 Statistical analysis

All the results were expressed as mean ± *SD*. Statistical analysis was evaluated by Student's *t* test, and a *p*‐value < .05 was believed as statistically significant difference.

## 3 RESULTS

### 3.1 Solubility studies

The solubility of OPC in various components was presented in Table 1. Since OPC showed its high solubility in aqueous vehicles compared to other oil‐type liquids, the distilled water was picked as a medium to load OPC. Commonly, several oils, solid lipids, and surfactants exhibited poor drug solubility that made the drug majority in the aqueous phase, and the excipients which had lowest solubility for OPC were selected to wrap the inner phase. Based on Table 1, OPC performed similar low dissolvability in various oil, solid lipid, and surfactants, so the solubility of OPC was not the only criteria during the selection. Other subsequent experiments were carried out to further optimize the model.

**TABLE 1:** The solubility of OPC in various vehicles

Vehicles	Solubility of OPC (mg/g)
Aqueous buffer solution (pH 5.0)	272.35 ± 7.18
Oils and solid lipid	
Corn oil	0.75 ± 0.06
Soy oil	0.88 ± 0.05
Sunflower oil	0.73 ± 0.04
Rapeseed oil	0.62 ± 0.08
MCT	0.94 ± 0.12
GML	0.02 ± 0.02
GMS	0.02 ± 0.02
Surfactants	
PGPR	0.06 ± 0.01
B‐18B	0.03 ± 0.02
Span 60	0.08 ± 0.02
Span 80	0.09 ± 0.02
Tween 20	0.06 ± 0.02
Tween 60	0.05 ± 0.02
Tween 80	0.05 ± 0.02

### 3.2 Formulation optimization primary W/O emulsions

#### 3.2.1 Primary W/O emulsions

**FIGURE 1:**
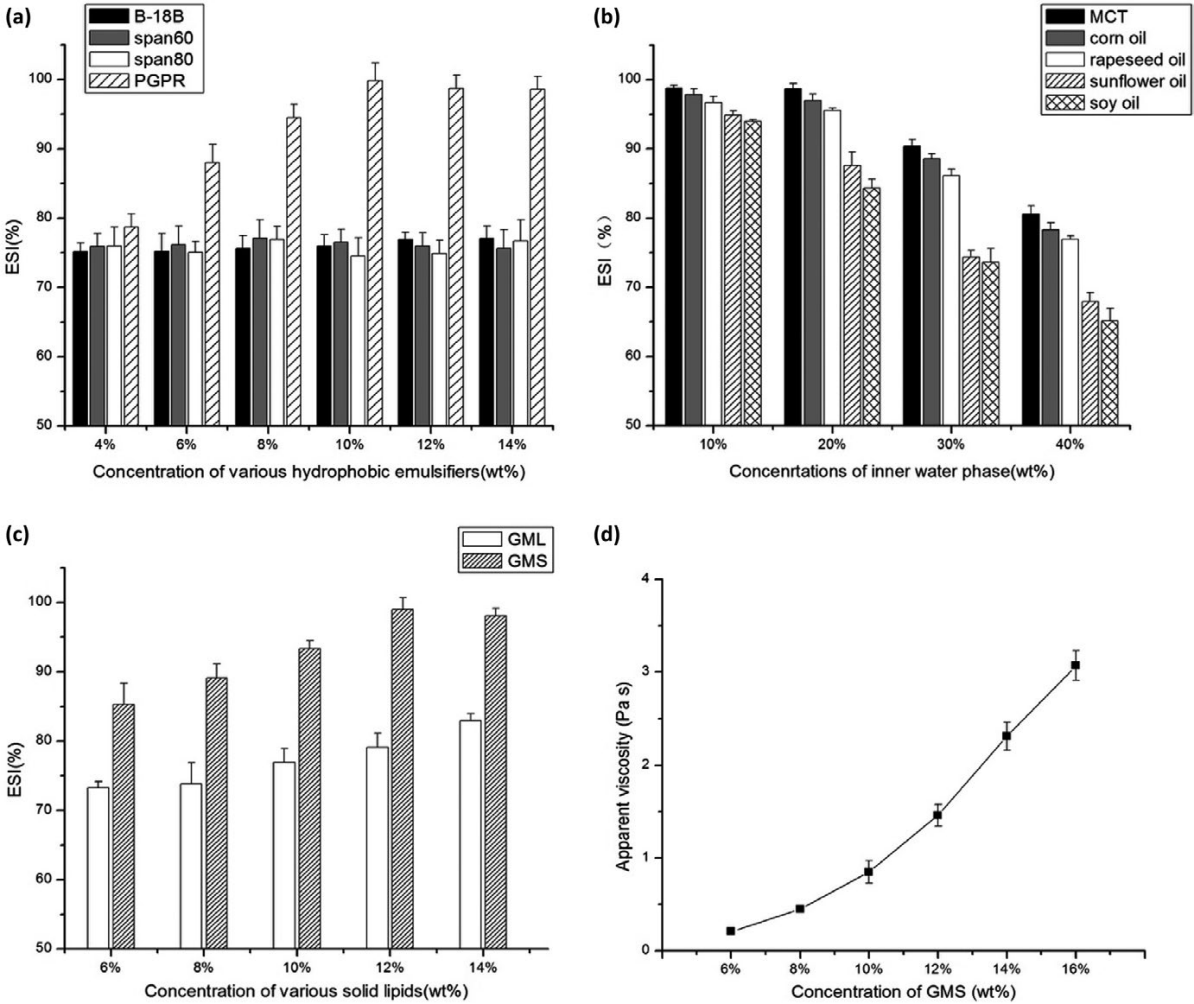
(a) Effect of hydrophobic emulsifier types and their concentrations (wt%) on emulsion stability index (ESI) of primary W/O emulsions. (b) Effect of oil types and concentration of inner water phase (wt%) on ESI of primary W/O emulsions. (c) Effect of solid lipid types and their concentrations (wt%) on ESI of primary W/O emulsions. (d) Effect of concentration of GMS crystals (wt%) on viscosity of primary W/O emulsions. Values are expressed as mean ± *SD* (*n* = 3)

The optimization for primary W/O emulsions was done by calculating ESI with selected hydrophobic surfactants during incubation at 65°C for 2 hr. To quantify the effect, different concentrations of separate hydrophobic samples were conducted to compare the emulsifying properties. As shown in Figure 1a, the ESI of the emulsion decreased in the order PGPR > Span60 > Span80 > B‐18B, and PGPR exhibited extraordinary ESI in the selection of hydrophobic surfactants. Frankly, PGPR has been proven to be one of the most powerful emulsifiers which could stabilize W/O emulsions structure the high emulsifying characteristic was ascribed to the great water‐binding of long hydrophilic polyglycerol chain (Marquez, Medrano, Panizzolo, & Wagner, 2010). The emulsifying abilities of various concentrations of PGPR were also compared in Figure 1a. 4 wt% of PGPR showed 79.73 ± 2.45% ESI, whereas 10 wt% of PGPR could reach over 99% ESI. The results indicated the ESI of primary W/O emulsions gradually increased and saturated at 10 wt% of PGPR. Hence, the 10 wt% PGPR was picked to optimize the stability of emulsion.

After determining the type of hydrophobic surfactant and its concentration, various oils were investigated to formulate a stable emulsion. Figure 1b showed emulsions formulated with medium‐chain triglycerides (MCT) performed most stability compared with other oils, and the ESI of emulsions degraded in the order of MCT > corn oil > rapeseed oil > sunflower oil > seed oil. The results indicated that MCT was the best candidate to formulate emulsion along with PGPR. Furthermore, the impact of inner phase concentration on ESI of primary W/O emulsions was tested as well. As Figure 1b showed, gradually raising inner the water phase concentration of primary W/O emulsions from 10 wt% to 40 wt% resulted in a decrease of ESI. Regarding MCT, ESI was examined to be 98.79 ± 1.62% under 20 wt% of inner water phase, whereas 30 wt% of inner water phase would drop ESI to 87.92 ± 0.54%. The higher concentration of inner phase it reached, the lower ESI it got (*p* < .05). Results showed that concentration of inner water content over 30 wt% will cause phase separation at 65°C. A similar case had been reported when researchers studied on multiple lipid particles (Zhao et al., 2015). The apparent viscosity of formulations exhibited a decreasing trend regarding to elevation of inner water phase. This finding further proved phase separation was more likely to occur.

In our proposal, solid lipids such as GMS and GML could be both utilized as a solidifying agent during formulations. As shown in Figure 1c, though the stability of primary W/O emulsions revealed an increasing trend when the concentration of solid lipids was raised from 6 wt% to 14 wt%, the same proportion of GMS could achieve higher ESI. Hence, different concentrations of GMS (6 wt%, 8 wt%, 10 wt%, 12 wt%, 14 wt%, and 16 wt%) for primary W/O emulsions were optimized by viscosity. From Figure 1d, with the increment of GMS in outer oil phase, the viscosity showed a significant increasing trend. When GMS concentration reached 12 wt%, the viscosity of OPC‐SDEDDS reached 1.42 ± 0.12 Pa·s. Hence, the formulation was obtained by semisolid or solid status and displayed better chemical/physical stability. This may be due to the fact that in the emulsion, the movement of the inner water droplet could be suppressed by high viscous outer oil phase (Schuch et al., 2013). Consequently, the addition of 12 wt% GMS could be amenable to the stability of OPC‐SDEDDS.

#### 3.2.2 Optimizing the hydrophilic emulsifier

**FIGURE 2:**
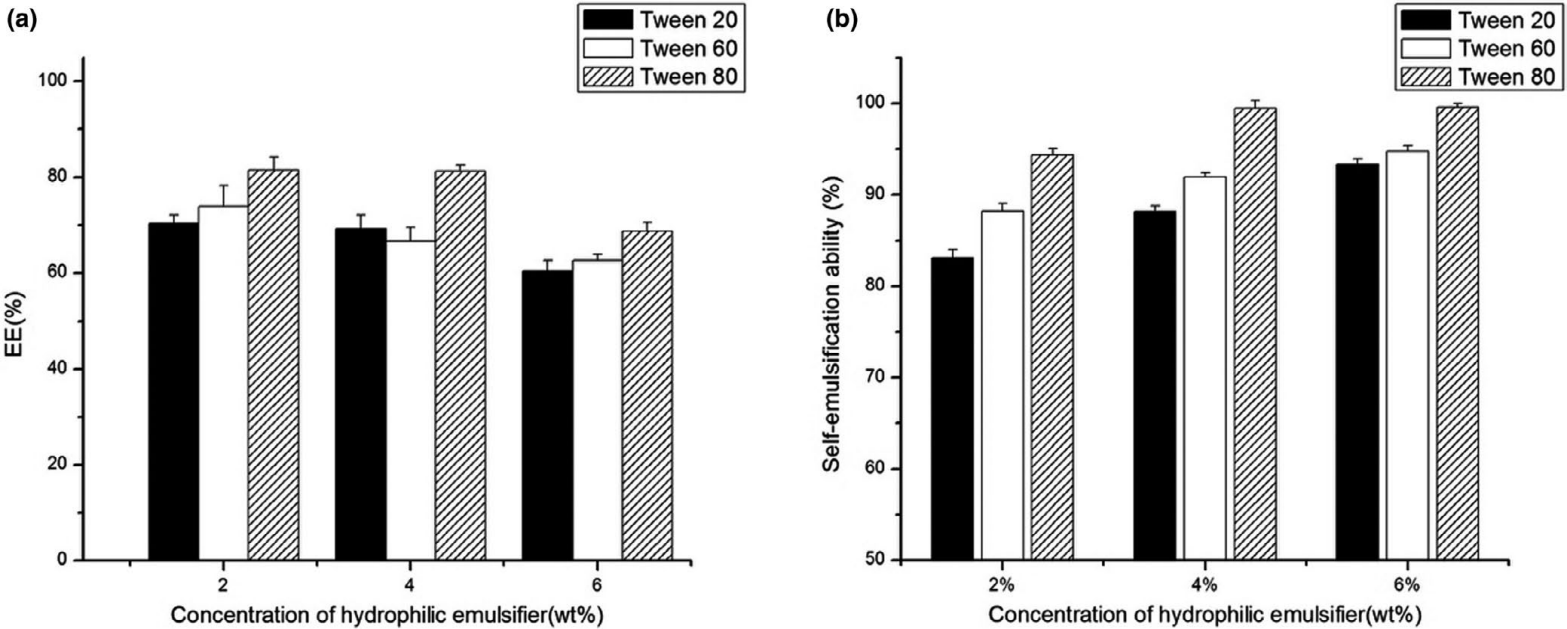
(a) Effects of types hydrophilic emulsifiers and their concentrations on the encapsulation efficiency (EE) of OPC‐SDEDDS. (b) Effects of types hydrophilic emulsifiers and their concentrations on the solubility of OPC‐SDEDDS. Values are expressed as mean ± *SD* (*n* = 3)

Oligomeric proanthocyanidin‐self‐double‐emulsifying drug delivery system was developed by employing different HLB‐valued hydrophilic surfactants, and the optimal formulation was based on the EE and self‐emulsifying ability of OPC‐SDEDDS. Different concentrations (2 wt%, 4 wt%, and 6 wt%) with Tween 20, Tween 60, and Tween 80 were picked as hydrophilic emulsifiers. The EE and self‐emulsification ability were measured, respectively. As shown in Figure 2a,b, when Tween 20 was opted as emulsifier, the highest EE for OPC‐SDEDDS and self‐emulsification ability were 70.52 ± 2.63% and 93.32 ± 1.74%, respectively. Meanwhile, if Tween 60 was selected, the formulation revealed similar EE and self‐emulsification ability as 73.31 ± 2.66% and 94.77 ± 2.04% measured as highest values individually. Therefore, Tween 80 was considered the most effective emulsifier—the greatest values of EE and self‐emulsification ability could be obtained as 80.13 ± 2.86% and 98.59 ± 0.92% separately. Besides, Tween 80 displayed high EE and self‐emulsification ability in different concentrations. It was observable that regardless of the type of hydrophilic surfactant, the EE decreased as the concentration of surfactant increased. Contrarily, the self‐emulsification ability of OPC‐SDEDDS was positively correlated with the concentration of the surfactant. The previous research had proposed that the coalescence of thin liquid film from high concentration of hydrophilic surfactants could separate the internal droplets and globule surface, which speeded up the release of inner droplets (Pays, Giermanska‐Kahn, Pouligny, Bibette, & Leal‐ Caldron, 2001). In addition, toxicological ability was raised when increasing hydrophilic emulsifier. Hence, Tween 80 with 4 wt% concentration value was picked for further analysis.

### 3.3 Morphological characterization of optimum formulation

**FIGURE 3:**
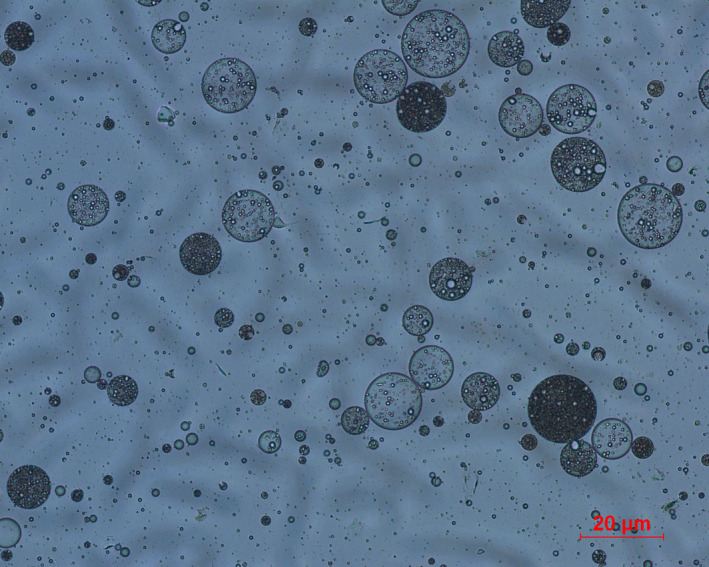
Microscopy image of OPC‐SDEDDS in aqueous media. Scale bars, 20 μm

Oligomeric proanthocyanidin‐self‐double‐emulsifying drug delivery system, a reddish brown solid, was able to self‐emulsify into homogeneous emulsions with a pellucid representation when it was dispersed into aqueous medium. The optimal formulation did not show the phenomenon of phase separation and drug precipitation though after 6 hr. The OPC‐SDEDDS preparing was diluted into distilled water with tender stirring, and microscopy photographs were taken to analyze double emulsions structure. As shown in Figure 3, the micrograph indicated that the small dispersed inner water droplets were well‐wrapped with external oil layer, which was in conformity to the features of double emulsions. These results indicated that SDEDDS could well form W/O/W double emulsions through dilution with dispersion medium at mild agitation. Note that most of double emulsions shape observed were spherical.

### 3.4 Solid‐state characterization of OPC‐SDEDDS

**FIGURE 4:**
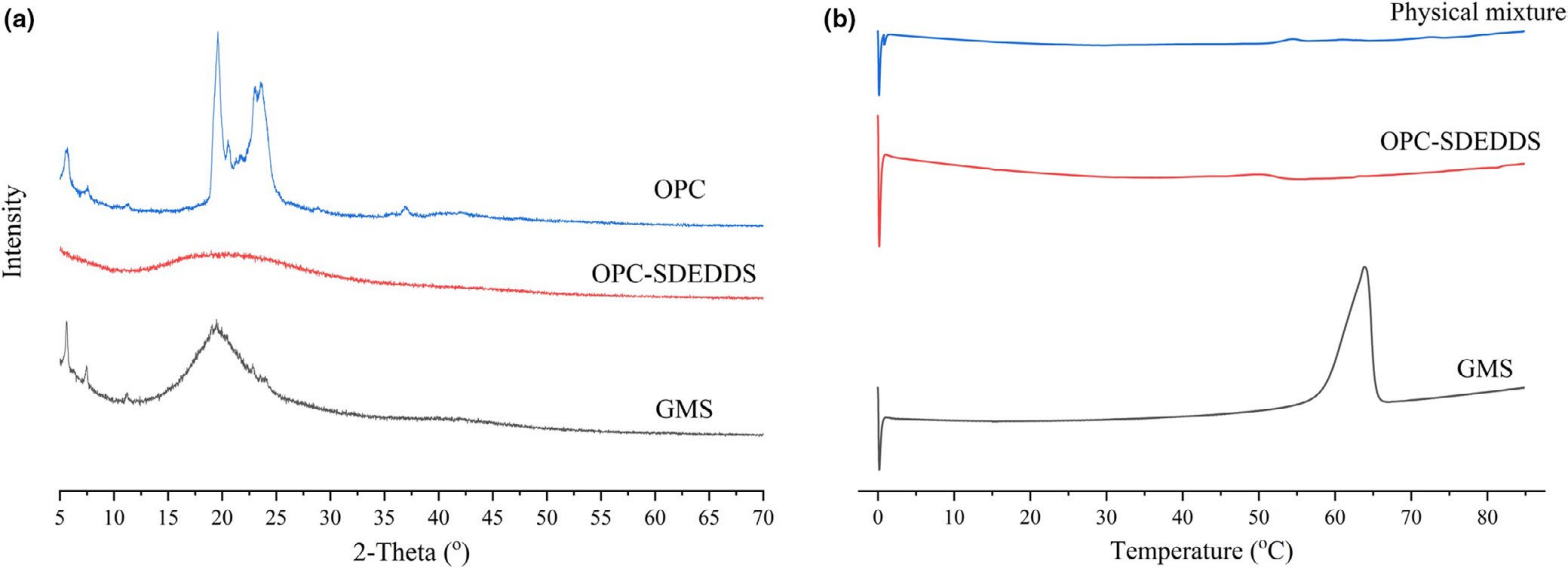
(a) X‐ray diffraction results of GMS crystals, solid OPC‐SDEDDS, and OPC. (b) Differential scanning calorimetry (DSC) curves of GMS crystals, solid OPC‐SDEDDS, and the physical mixture of SDEDDS

The physical state of solid OPC‐SDEDDS was characterized by XRD and DSC, respectively. Figure 4a showed the X‐ray diffraction patterns of different samples ranged from 5° to 70°. It could be illustratable that the peak of characteristic high‐energy diffraction of OPC occurred between 5° and 30°, which meant drug was present as a crystalline material. The same crystalline characteristics were found in the spectrum of GMS crystal. However, the characteristic diffraction peaks of OPC disappeared in the spectrum of OPC‐SDEDDS, suggesting that crystal OPC was soluble and amorphous in inner water phase. Kanuganti, Jukanti, Veerareddy, and Bandari (2012) also reported the phenomenon that characteristic peaks of the active vanished after encapsulation. Meanwhile, the reduced crystallinity of the GMS crystals (the peaks did not completely disappear) also implied that the GMS crystals were still in β‐form after the incorporation of the active in SDEDDS. The β‐form normally had a dense crystalline parking and highly ordered fatty acid chains, and it could improve the stability of emulsions (Fathi, Varshosaz, Mohebbi, & Shahidi, 2013).

Typical DSC heating/cooling curves were shown in Figure 4b. In this article, GMS crystals and physical mixture of SDEDDS were used to compare with solid SDEDDS. The GMS heating curve showed a significant transition at the peak of 63.85°C, which could due to the melting of GMS crystals (Yoon, Chung, & Han, 1999). For the solid SDEDDS, a characteristic endothermic peak (50.35°C) appeared, presenting a lower melting point than the bulk lipid. It could be contributed from uniform distribution of GMS in the liquid oil. The unmissing peak implied the crystals of GMS in SDEDDS remain β‐form, and it was consistent with XRD in this study. In addition, two peaks appeared in the SDEDDS physical mixture graph which suggested that melting of different components in the mixture.

### 3.5 DPPH radical scavenging of OPC‐SDEDDS

**FIGURE 5:**
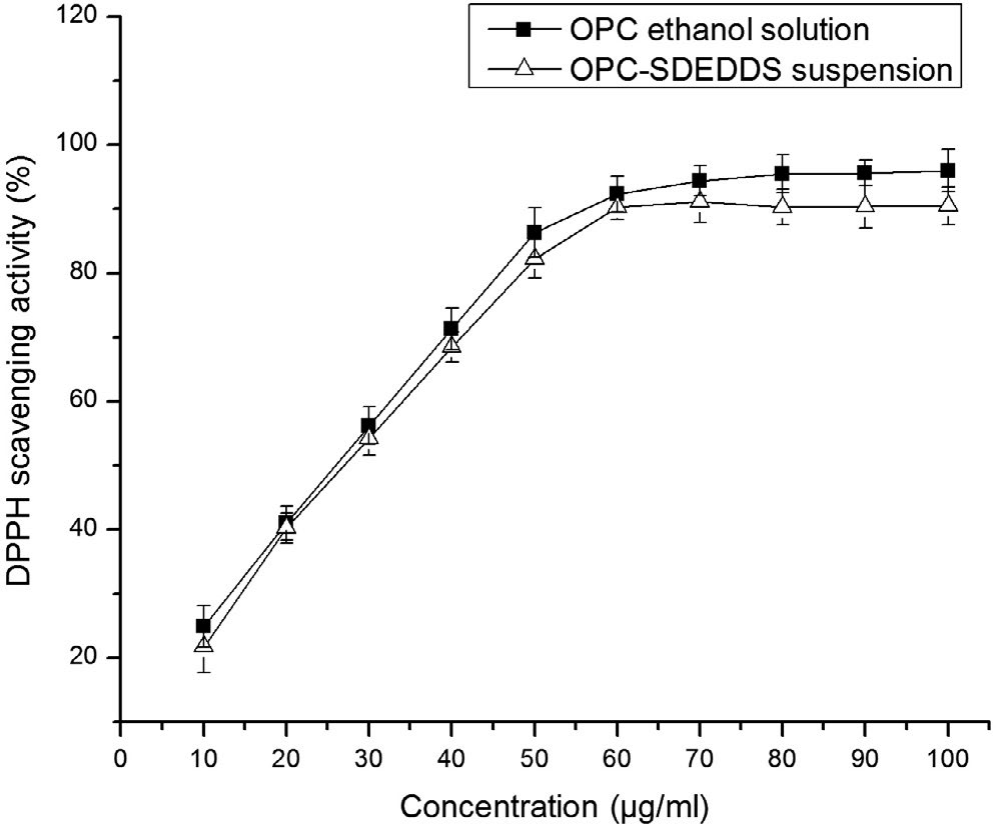
2,2‐Diphenyl‐1‐picrylhydrazyl (DPPH) radical inhibition

1,1‐diphenyl‐2‐picrylhydrazyl colorimetric assay is a common way for evaluating functional activity of different antioxidants (Meda, Lamien, Romito, Millogo, & Nacoulma, 2005). In this study, the antioxidant ability of solid OPC‐SDEDDS was measured by DPPH assay, setting up pure OPC as positive control. Figure 5 exhibited the percentage DPPH radical scavenging activity of pure OPC and OPC‐SDEDDS. The curves showed DPPH free radical scavenging activity was positively correlated with OPC concentration. No obvious change (*p* > .05) had been observed in the scavenging activity of pure OPC and OPC‐SDEDDS. It could be concluded that from the DPPH test, pure OPC did not loss biological activity from encapsulating in solid SDEDDS.

### 3.6 Stability of OPC‐SDEDDS

#### 3.6.1 Illumination stability

**FIGURE 6:**
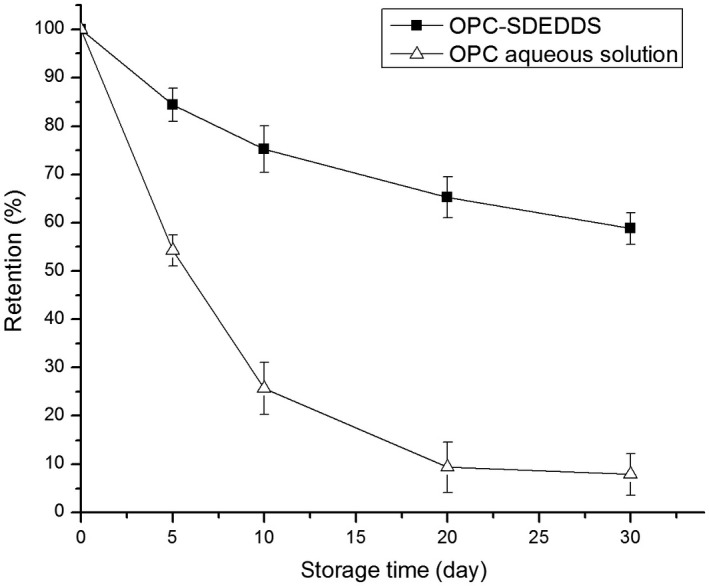
OPC‐SDEDDS and OPC solution stores under light or in dark. Values are expressed as mean ± *SD* (*n* = 3)

To evaluate the illumination stability of OPC‐SDEDDS, OPC content of OPC‐SDEDDS was studied and stored under natural light exposure. As Figure 6 showed, after storing OPC‐SDEDDS under light conditions over 30 days, the retention of OPC was 62.83 ± 2.64%, which performed significantly higher than that of pure OPC control solution (8.34 ± 1.33%). The best result could be observed as OPC‐SDEDDS preserved in the dark environment, where the OPC content could remain 71.32 ± 3.13% after 30 days. Thus, these results showed that the illumination stability of OPC improved after loaded by SDEDDS. It may because OPC was a photosensitive substance, and the light was blocked by the encapsulating lipid particles. In addition, OPC was mainly distributed in the internal water phase, and the outer oil phase might act as a shell to protect drug molecules from oxidation and degradation (Kim & Kim, 2014; Lorenceau et al., 2005).

#### 3.6.2 Storage stability

**FIGURE 7:**
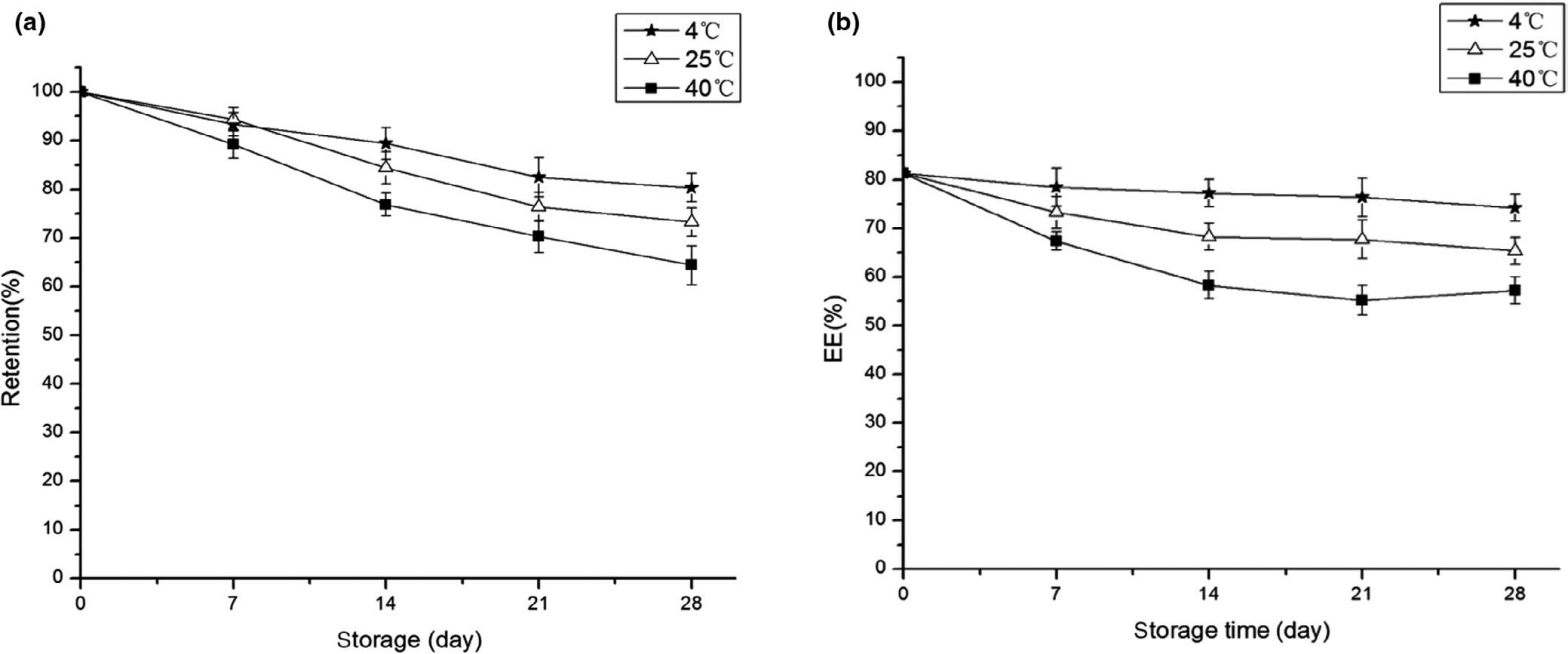
(a) The retention and (b) encapsulation efficiency of OPC‐SDEDDS in different temperatures. Values are expressed as mean ± *SD* (*n* = 3)

Self‐double‐emulsifying drug delivery system is an unstable system in thermodynamics that prone to be instability such as sedimentation and coalescence during stored for a long period. From the external inspection of OPC‐SDEDDS, it still remained a brown‐red uniform appearance and there was little unstable phenomenon after 28 days of storage under different temperature. The stability studies were displayed in Figure 7, under three conditions up to 28 days. The retention rate and encapsulation efficiency of OPC in SDEDDS were all show a slow decline trend. Besides, low temperature (4°C) environment was more conducive to preserve OPC‐SDEDDS for a long period, the retention rate of OPC under this condition was around 80%, and there were minor changes in encapsulation efficiency.

However, it was interesting that the encapsulation efficiency of OPC‐SDEDDS did not decrease as expected during the 4th week of storage at 40°C—instead, it increased. The phenomenon was due to the stability of OPC is poor at a higher temperature, and unwrapped OPC in SDEDDS became inactivated. It resulted in an increase in the encapsulation efficiency of the calculated OPC‐SDEDDS.

### 3.7 In vitro release studies

**FIGURE 8:**
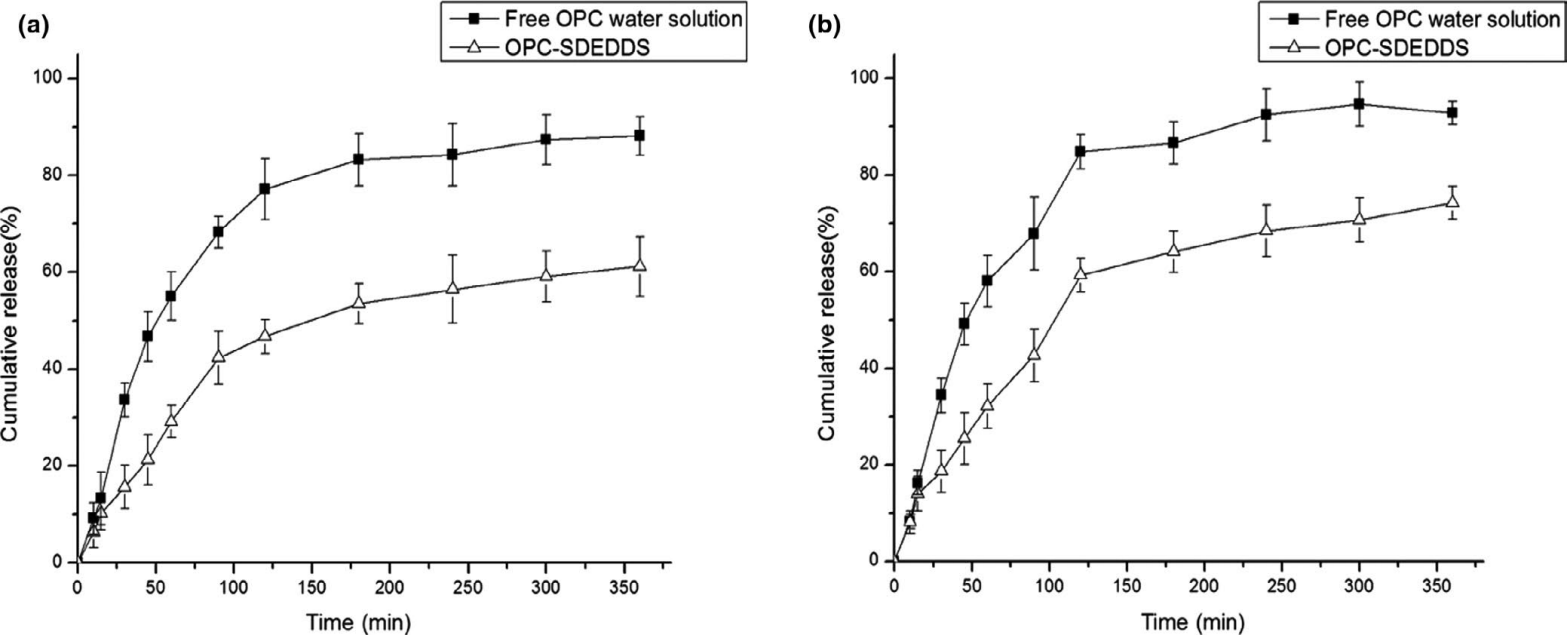
Cumulative amount of OPC released from SDEDDS and water solution in (a) simulated gastric fluid and (b) simulated intestinal fluid over a period of 6 hr. Values are expressed as mean ± *SD* (*n* = 3)

In this research, the drug molecular size was dramatically lower than the selected molecular weight cutoff of the dialysis membrane; thus, drug would be able to pass through the membrane of dialysis bag. The cumulative in vitro release curves of OPC‐SDEDDS suspension and OPC solution are depicted in Figure 8, where in the release medium of Figure 8a was simulated under gastric fluid (SGF) and Figure 8b under intestinal fluid (SIF). The release of OPC from OPC‐SDEDDS suspension in SGF was fitted to the Weibull equation model as: log [−ln (1 − *Q*)] = 0.7566 log *t* – 1.8597 (*r* = .9707). Besides, the release in SIF could be evaluated as: log [−ln (1 − *Q*)] = 0.7817 log *t* − 1.7983 (*r* = .9801), where *Q* and *t* stood for cumulative drug release and time (min), respectively. The slope value *k* (also known as shape factor) could be used to evaluate the mechanism of drug transport from the inner aqueous phase. The values of shape factor *k* for OPC from OPC‐SDEDDS suspension in SGF and SIF were 0.7566 and 0.7817 distinctively. Therefore, Fickian diffusion and Case‐II transport (value in the range 0.75 < *k* < 1) could be regarded as the main mechanism of drug release from OPC‐SDEDDS (Tatini et al., 2015).

Moreover, the release rate of OPC from ethanol was much faster both in SGF and in SIF, and over 80% of the drug was released within 3 hr. On the contrary, merely 53.52% of OPC was released from SDEDDS suspension in SGF and 64.11% in SIF within same period. There were two potential mechanisms that might contribute to the sustained release of drug from SDEDDS suspension. One is because the thin‐solid film that separated inner droplets and the globule surfaces had coalesced. The other mechanism could be the diffusion and/or permeation of drug across the outer oil phase. In other words, since most of drugs were dissolved in inner aqueous phase, the external oil layer automatically formed as a barrier for the diffusion of OPC (Genty et al., 2003). In addition, comparing the release curves in two different media, OPC in SIF could get a relatively higher cumulative release rate since it was more stable in the alkaline environment. While OPC was in SGF (pH 1.2), the structure of small amount OPC had changed and the content density reduced. There was another phenomenon observed in the graph revealing the beginning burst of release phase, which was followed by a lag phase. The burst release phenomenon could be the low distribution of drug molecules in outer oil phase and hydrophilic surfactants (Hu, Wang, Ma, & Xia, 2016). Afterward, the transport of OPC across the outer oil phase into the aqueous dialysis medium affected the release rate.

### 3.8 In vitro lipid digestion

**FIGURE 9:**
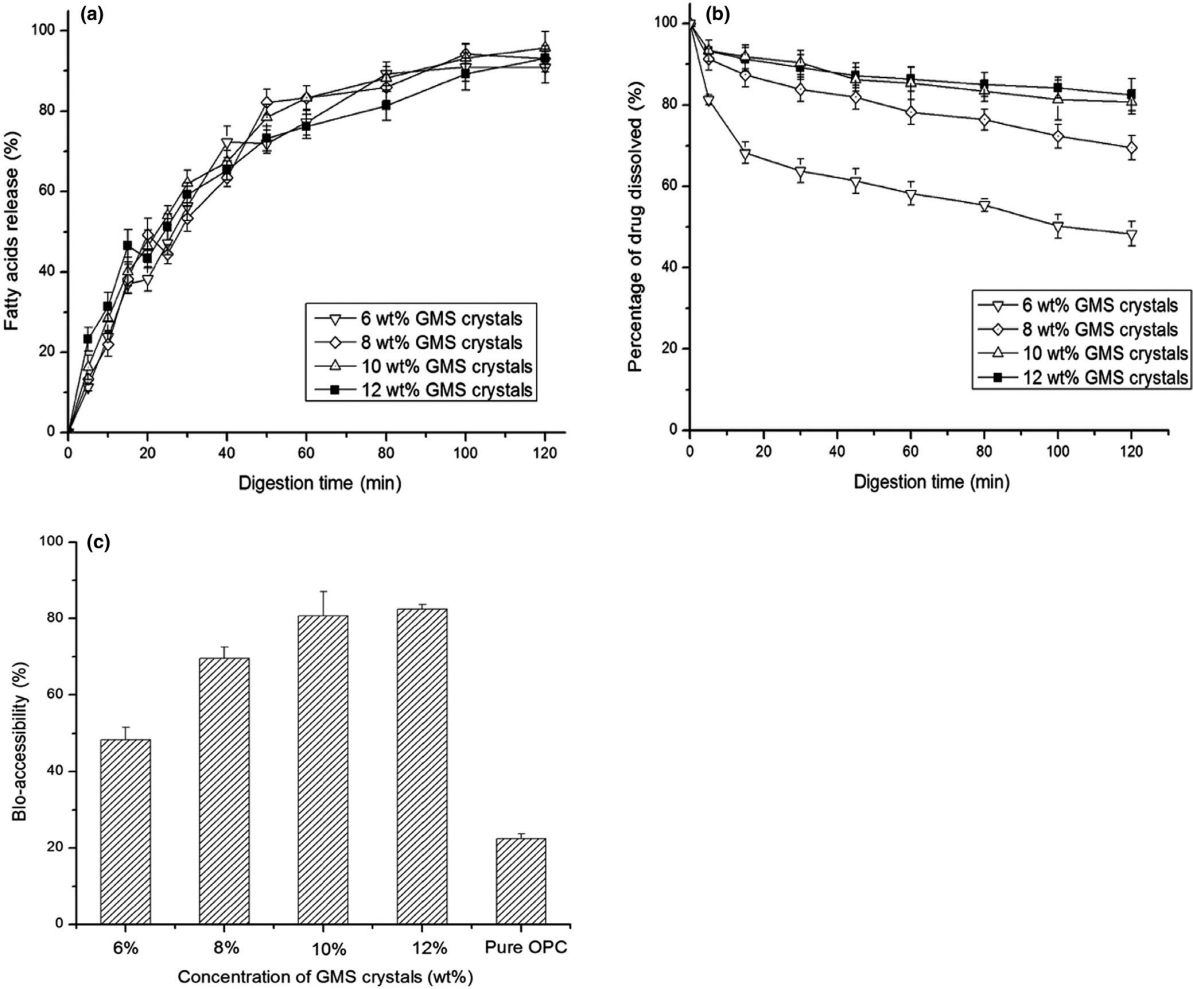
(a) Amount of fatty acids released from OPC‐SDEDDS containing different GMS crystal concentrations as determines using a pH‐stat titration technique. (b) Percentage of OPC that remains dissolved after the digestion of the OPC‐SDEDDS containing different GMS crystal concentrations in SIF. (c) Bioaccessibility of the OPC‐SDEDDS containing different GMS crystal concentrations and the pure OPC powder. Values are expressed as mean ± *SD* (*n* = 3)

In this experiment, when the droplets from OPC‐SDEDDS containing different concentrations of GMS (6 wt%, 8 wt%, 10 wt% and 12 wt%) passed through the gastric digestion, there was little change observed by microscope. However, when samples were at the end of intestinal digestion, increase of droplets sizes was remarkable. This indicated that droplets of OPC‐SDEDDS still carried on the morphological and structural integrity in SGF environment, then been hydrolyzed, and digested in SIF. In addition, free fatty acids were formed by the degradation of ether‐based lipid elements during the digestion in SIF (Hur, Lim, Decker, & McClements, 2011). In PH‐stat titration method, the amount of NAOH used could indicate the release of fatty acids from pancreatic lipase. Figure 9a showed the ratio of free fatty acids released from the samples and recorded as a function of time. Comparable trends could be viewed when the concentration of GMS crystal increased from 6 wt% to 12 wt%. As expected, the GMS crystal was vulnerable to digestion proved by the fatty acid titration images. Likewise, other ether‐based surfactants (PGPR and Tween 60) were digestible as well (Mohsin, 2012). Additionally, though the initial (0–50 min) rate of reaction was fast, it later dropped at next 70 min. Generally, lipid formulation for oral administration should be capable of retaining activity in solubilized form all over the gastrointestinal tract. Nevertheless, the capacity of solubilization was susceptible to lipid digestion (Ozturk, Argin, Ozilgen, & McClements, 2015). Thus, the impact of digestion on active solubilization was recorded in SIF environment.

From Figure 9b, the solubilization capacity increased when GMS crystal concentration rose from 6 wt% to 12 wt%. After 2 hr of digestion, 82.45% of OPC reserved in the solubilized form with formulations including 12 wt% of GMS crystals. When the concentration of GMS crystals in formulations was lower (6 wt%–8 wt%), the precipitation of OPC reacted more quickly, and the reason caused this was the lower amount of lipid droplets/mixed micelles formed after lipid digestion (Salvia‐Trujillo, Qian, Martín‐Belloso, & McClements, 2013). Some researches demonstrated that the multilayer liquid crystalline phases on the surface of droplets were generated from the lipolysis products loaded with active. These phases were detached from the surface of droplets, interacted with bile salts to form micelles, and eventually absorbed by intestinal cells (Fatourosa, Bergenstahl, & Mullertz, 2007). Thus, the increase in solubilization was related to the increase of lipid droplets/mixed micelles which were able to solubilize the OPC released from the SDEDDS. Based on this, protection of OPC against digestion‐mediated precipitation was observable when the formulations containing high volume of GMS crystals.

Figure 9c depicted the bioaccessibility of pure OPC and OPC‐SDEDDS (containing different ratios of GMS). Significant improvement of bioaccessibility was shown after loading OPC to SDEDDS, and the bioaccessibility of OPC‐SDEDDS containing different concentrations of GMS was all over 48%, yet pure OPC merely reached 22.47% bioaccessibility. In addition to the excipients in OPC‐SDEDDS which played a critical role in improving OPC absorption explained in above paragraph, there was another mechanism could explain the increased bioaccessibility of OPC in SDEDDS. The emulsion formed by SDEDDS in SGF maintained the integrity and morphology of structure before getting to the SIF, which made OPC mainly solubilized in the internal aqueous phase and protected it from enzymatic degradation.

## 4 CONCLUSION

In this study, a solid SDEDDS loaded with OPC was proposed and optimized to improve the bioavailability of active ingredient. The formulated OPC‐SDEDDS could self‐emulsify to W/O/W double emulsions via dispersion into aqueous media. Solid‐state characterization was performed by DSC and X‐ray powder diffraction, and the result confirmed that OPC existed in the OPC‐SDEDDS as the amorphous state. Additionally, the selected formulation displayed sustained release profile in vitro and was suitable for storage in a dark and low temperature condition. OPC‐SDEDDS had the same antioxidant activity as OPC, which indicated that the antioxidant capacity of ingredient was not degraded after loaded by SDEDDS. More than 80% of OPC in SDEDDS were solubilized after 2 hr of digestion with the formulations containing 12 wt% GMS crystals. The bioaccessibility of OPC from SDEDDS was dramatically higher than pure OPC. Last but not least, the optimized solid SDEDDS was proved reliable through experimental evaluation, which could be used for oral delivery of water‐soluble drug (e.g., OPC).
